# Improving Accuracy and Timeliness of Nursing Documentation of Pediatric Early Warning Scores

**DOI:** 10.1097/pq9.0000000000000278

**Published:** 2020-03-25

**Authors:** Nathan P. Dean, Jenhao J. Cheng, Ian Crumbley, Jennifer DuVal, Eliana Maldonado, Emanuel Ghebremariam

**Affiliations:** From the * Department of Pediatrics, Children’s National Health System, The George Washington School of Medicine; † Department of Quality and Patient Safety, Children’s National Health System; ‡ Department of Information Technology, Children’s National Health System; § Department of Medical Nursing, Children’s National Health System; ¶ Department of Surgical Nursing, Children’s National Health System; ‖ Department of Performance Improvement, Children’s National Health System

## Abstract

**Introduction:**

Accurate and timely documentation of pediatric early warning scores (PEWS) by the bedside nurse into the electronic health record (EHR) is important to promote early identification of patients in stages of deterioration. Through the implementation of a PEWS calculator embedded in the EHR, we hope to improve the accuracy of the recorded score and reduce the time between vital sign collection and final documentation in the EHR.

**Methods:**

Identification of the highest PEWS value in the 24 hours before all unplanned transfers or rapid response activations without a transfer occurred between the period November 1, 2013, through December 31, 2016. The accuracy of the calculated cardiac or respiratory subscore based on heart rate or the respiratory rate at the time of PEWS calculation was determined. We tracked the calculation of the time to chart via the difference between nursing documentation of PEWS compared to vital sign collection. Before September 3, 2015, PEWS was calculated mentally by the bedside nurse; afterward, the nurse entered the unique PEWS features into the EHR, and the EHR automatically calculated PEWS.

**Results:**

This study evaluated 2,409 PEWS scores, 1,411 before and 998 after initiation of the PEWS calculator. Accuracy before the EHR calculator was 71%, and the median time to document was 55 minutes. Following the implementation of the calculator, no scores were incorrectly calculated too low, and the median time to document was 20 minutes.

**Conclusions:**

Transition to an EHR-based PEWS calculator eliminated inaccurately low PEWS values and reduced time to document.

## Abstract 2

Supplemental Digital Content is available in the text.

## INTRODUCTION

Early recognition and identification of deteriorating patients are critical to prevent further progression. Pediatric early warning scores (PEWS) utilize vital signs and components of the routine bedside nursing examination to calculate a score that may suggest a patient is at risk for deterioration. Many institutions have adopted PEWS, and based on the calculated score, PEWS may trigger an evaluation by a Rapid Response Team (RRT). Accurate scores and timely documentation are essential for these tools to be effective. Scores calculated manually are at risk for calculation errors by the documenting nurse. A PEWS erroneously calculated too low based on the recorded vital signs may result in a missed mandatory escalation and further deterioration. Additionally, multiple steps are required collecting vital signs and physical examination findings, manually calculating a score, and entering examination findings into the EHR that might require different fields of entry. This process prolongs the time to enter a patient’s PEWS, delaying the recognition of deterioration.

Utilization of electronic health records (EHRs) has significantly increased since the passing of the Affordable Care Act in 2009.^1^ One of the goals of the EHR is to improve documentation. When compared with paper charting, documentation utilizing the EHR is more thorough^2–4^ and provides better structure.^5^ Other studies looking at the time saved with EHR documentation have shown mixed results, but self-reporting limits interpretation of the results.^6^ Despite these reported benefits, other studies identify unintended consequences resulting in a negative impact of EHRs on nursing workflow, inconsistent accessibility of information, and flowsheet design mismatches to nursing work.^7,8^

One advantage of the EHR is to simplify charting and avoidance of manual calculations by the bedside nurse by allowing the nurse to enter the fields and allow the EHR to perform the calculation. This functionality should simplify documentation and improve accuracy and timeliness when documenting. The objective of this study is to evaluate the effect of the implementation of an EHR-based calculator on accuracy and timeliness of documentation of PEWS in patients demonstrating evidence of deterioration.

## METHODS

Children’s National Hospital utilizes a modified version of the Brighton PEWS^9^ to promote the identification and rescue of deteriorating patients. PEWS of 0-2 result in normal monitoring, a score of 3 requires notification of a physician and re-evaluation within 2 hours, a score of 4 or individually scoring a 3 in one category requires a prompt bedside evaluation by the primary team, bedside RN and charge nurse, and a score of 5 or greater requires the bedside RN to initiate an RRT activation manually. The RRT is composed of a pediatric intensive care unit (PICU) fellow or nurse practitioner, a PICU charge nurse, and a PICU-based respiratory therapist. Before September 3, 2015, the collection of vital sign components of the PEWS was by a patient care tech and delivered to the bedside nurse or collected directly by the bedside nurse. The bedside nurse would mentally calculate the PEWS using the collected vital signs and the nurse’s clinical assessment and then later enter the total PEWS and each PEWS subscore (cardiac, respiratory, and behavior) into the EHR. After September 3, 2015, the EHR was changed so that the bedside nurse enters each component of the PEWS into the EHR, and the EHR automatically calculates the PEWS and prompts the bedside RN to engage in the appropriate escalation based on the score (**see Table 1, Supplemental Digital Content 1**, http://links.lww.com/PQ9/A176).

Since November of 2013, tracking and prevention of patient deterioration have been monitored by the Late Rescue Collaborative (LRC). The LRC is a multidisciplinary hospital committee with members from acute and critical care units which evaluates hospital escalation policies and reviews individual cases of deteriorations. During this time, the LRC has tracked all RRT activations and all unplanned transfers. The highest PEWS value in the 24 hours before all unplanned intensive care unit (ICU) transfers and all RRTs without transfer was recorded and evaluated for accuracy and timeliness by members of the LRC.

An automated daily report providing a list of patients with a change in bed space identified unplanned transfers, and a manual recording in an Excel spreadsheet tracked all RRT pager activations. The same identification, selection, and review of patients occurred preintervention and postintervention. Determination of accuracy occurred by chart review done by physicians and nurses participating in the LRC. For accuracy, the cardiac and respiratory subscores must meet the expectations based on the most recent set of recorded vital signs (**see Table 2, Supplemental Digital Content 2**, http://links.lww.com/PQ9/A176). The definition of timeliness is the interval between time vital signs are collected (reflected by the “Results Time” timestamp in the EHR) and when the bedside nurse enters the PEWS into the EHR and the PEWS becomes visible to all providers (“Valid From” timestamp). Determination of timeliness occurred via automated query of the “Results Time” and “Valid From” timestamps in the EHR during the study period.

The preintervention period was November 1, 2013, through September 2, 2015. The postintervention period was September 3, 2015, through December 31, 2016. We utilized statistical process control charts to demonstrate change following the implementation of the automatic calculator. The definition of special cause variation to detect a statistically significant process shift was a run of 8 or more consecutive points above or below the central line of the baseline period.^10^ We evaluated PEWS accuracy by a p-control chart. We utilized an Xmedian control chart and the Mann–Whitney tests to determine statistical significance between the preintervention and postintervention median time to chart PEWS. Finally, we tracked non-ICU arrests (defined as cardioversion, defibrillation, cardiopulmonary resuscitation, or intubation on the acute care unit) with a u-control chart starting on October 1, 2014.

This project was a Quality Improvement Initiative at Children’s National, and it does not constitute human subjects research. As such, it was not under the oversight of the Institutional Review Board.

## RESULTS

There were 1,411 charts reviewed before the introduction of the EHR PEWS calculator, and 998 charts reviewed after the introduction of the calculator. PEWS ranged from 0 to 9 in both groups, and both groups had a similar distribution of PEWS values (Table 1).

**Table 1.**
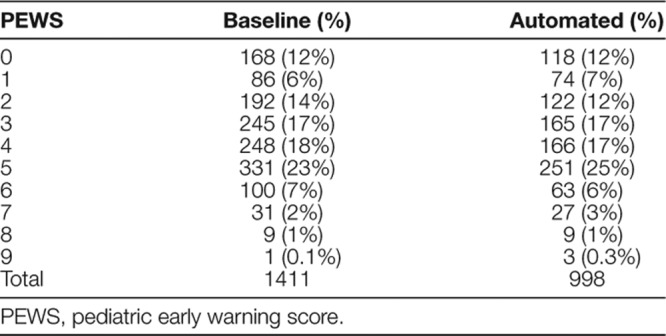
Breakdown of Pediatric Early Warning Scores

**Fig. 1.**
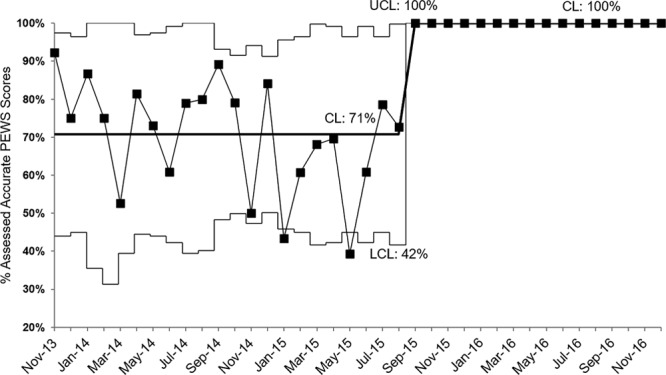
p-Control chart of pediatric early warning score accuracy. CL, center line; LCL, lower control limit; PEWS, pediatric early warning score; UCL, upper control limit.

The baseline mean accuracy of scores was 71%, ranging from 91% to 40%. Following the implementation of the PEWS calculator, accuracy was 100%, with no scores found to be inappropriately low based on concurrently charted vital signs (Fig. 1).

**Fig. 2.**
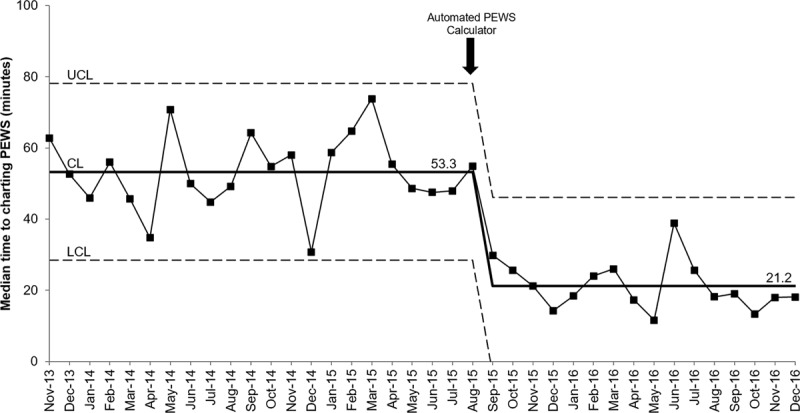
x-Chart of monthly medium time to chart pediatric early warning scores in minutes. CL, center line; LCL, lower control limit; PEWS, pediatric early warning score; UCL, upper control limit.

The average baseline median time to chart was 54 minutes ranging from 31 minutes in December of 2014 to 74 minutes in March of 2015. After the intervention, the average median time to chart was 20 minutes with all values below the previous median of 55 minutes and the range also reduce 37% from 43 (31–74 min) to 27 minutes (12–39 min) representing reduced variability in charting time after the intervention (Fig. 2).

Time to chart was further broken down by PEWS grouped into 3 categories. The first category was low, which included all values 0-2, correlating to PEWS values that did not generate a specific response by the provider team. The second group was medium, and includes PEWS of 3 and 4, correlating to increasing concern and notification of the primary team. The final group was high and included all PEWS of 5 or greater. This group was designated high due to the mandatory evaluation by the RRT when a PEWS is 5 or greater. All 3 categories demonstrated a significant reduction in time to chart after the introduction of the EHR calculator. Patients in the low category had the longest time to chart pre and post calculator (82 and 46 min) versus patients in the medium (45 and 13 min) and high categories (41 and 15 min) (Table 2).

**Table 2.**
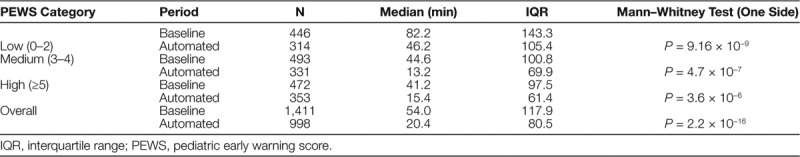
Comparison of Median Time to Chart Broken by Pediatric Early Warning Score Category by Mann–Whitley Test

**Fig. 3.**
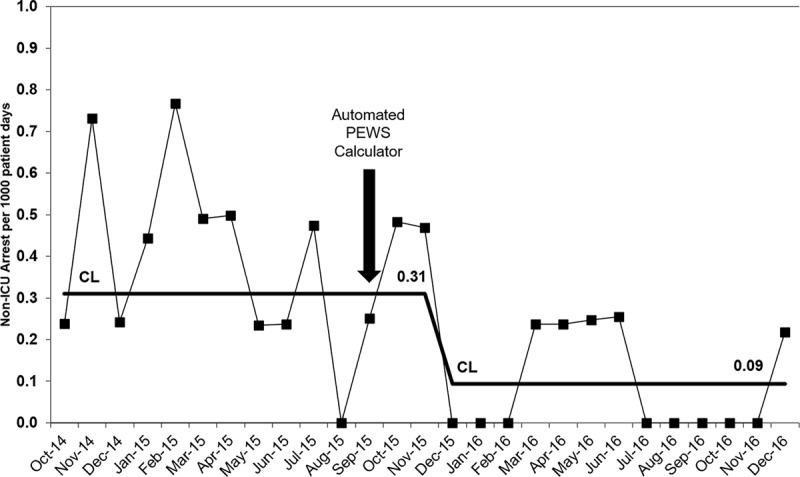
u-Control chart of non-intensive care unit arrest per 1000 nonintensive care patient days. CL, center line; ICU, intensive care unit; PEWS, pediatric early warning scores.

**Fig. 4.**
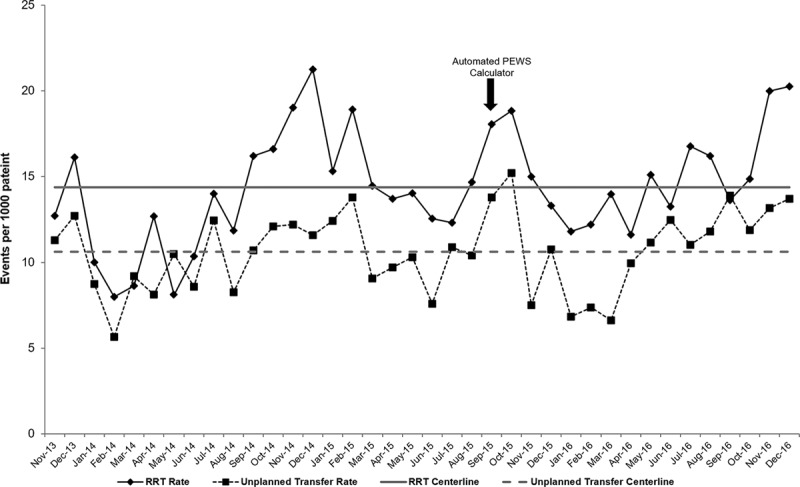
u-Control chart of rapid response activations and unplanned transfers per 1,000 nonintensive care patient days. CL, center line; ICU, intensive care unit; PEWS, pediatric early warning scores; RRT, rapid response team.

Non-ICU arrests demonstrated a centerline shift 2 months after the implementation of the calculator. The centerline rate dropped from 0.31 events per 1,000 patient days to 0.11 events per 1,000 patient days (Fig. 3). RRT activations showed no centerline shift after, but unplanned transfers did demonstrate a centerline shift eight months after implementation from 10.2 events per 1,000 patient days to 12.4 events per 1,000 patient days (Fig. 4). Tracking of compliance with mandatory RRT activations for PEWS of 5 or greater occurred from November 1, 2013, through December 31, 2015. Compliance with mandatory activation of the RRT before implementation was 87% and 94% after implementation.

## DISCUSSION

The EHR embedded PEWS calculator eliminated inaccurately scored low PEWS in patients requiring either unplanned ICU transfer or RRT activation by eliminating mistakes occurring during manual calculations. Additionally, the time between the collection of vitals and finalization of charting in the EHR was reduced by more than 50%. This project benefits from a simple modification of the EHR, resulting in improved workflow and decision support for the documenting bedside nurse.

Multiple studies have evaluated the relationship between PEWS and unplanned ICU transfers, RRT activations, or code blue activations, but only a few have evaluated the accuracy of PEWS documentation when compared to documented vital signs.^9,11–15^ As a result, this is one of the few studies to directly compare PEWS to concurrently charted vital signs to determine accuracy.

Gephart et al^8^ evaluated implementation of EHR tools and came 3 conclusions for successful implementation and to minimize unintended consequences: (1) have RNs involved in build, test, and rollout; (2) ensure representation of RNs from all care areas in system decision-making to help anticipate unintended consequences; and (3) involvement of RN administrator to advocate for RNs and identify super users. Although the hospital deterioration committee noted the identification of the problem (inaccurate scores and delayed documentation), the creation and implementation followed Gephart’s recommendations through the heavy involvement of the nursing staff on the creation of the automated calculator.

This project has a significant impact on patient safety. By eliminating inaccurately low scores, missed mandatory activations are less likely, and by reducing the time to chart, identification of elevated scores occurs sooner. The combination of these effects ensures a quick and appropriate evaluation of patients in the early-to-late stages of deterioration, to identify the appropriate rescue plan. A possible tradeoff to this project is the loss of awareness of how to calculate the PEWS by the bedside nurse, and thus reduce his or her understanding of the score itself. A calculation table is included for reference in the current electronic form to maintain nursing awareness of the score. Additionally, during periods of EHR downtime, the bedside nurse may struggle to calculate PEWS. Automation of the score may also impact the understanding of how each PEWS subcomponent relates to deterioration again, highlighting the importance of using PEWS as an aid and not an absolute decision point.

One limitation of this study is the inability to identify inaccurately elevated scores during our preimplementation phase. Before the implementation of the calculator, only information regarding the recorded heart rate, respiratory rate, and total subscore (cardiac, respiratory, or behavior) were available, rather than the nonvital sign components that might affect a subscore. As a result, if a score was higher than expected by the vital sign range, it was assumed that another feature (ie, poor perfusion for cardiac, or severe retractions in respiratory) resulted in the higher than expected score, and thus they were classified as “accurate” for our analysis. Whether or not the preimplementation documented vital signs were used by the bedside nurse to calculate a PEWS subscore cannot be determined, but serve as the best guess for vital signs at the time of PEWS assessment. A second limitation is that this study only evaluated patients with RRT activations or unplanned transfers. As a result, stable acute care patients may not experience the same benefits in accuracy and more timely documentation. It was felt that demonstrating improvement in patients showing signs of deterioration was the primary purpose, and thus the reason to study this population. Finally, we are unable to link the centerline shift to the implementation of the automated PEWS EHR calculator. During the study period, the implementation of other projects^16^ to reduce non-ICU arrests may have influenced the centerline shift. It is unclear how much of that reduction was due to the automated calculator.

## CONCLUSION

Implementation of an EHR-based automated PEWS calculator was a simple intervention that resulted in the elimination of inaccurately low PEWS values and earlier documentation in patients requiring RRT activations or unplanned ICU transfers. Although other interventions to promote patient safety occurred during this time, a reduction in non-ICU arrests was seen shortly after implementation and sustained. The addition of decision support and ease of documentation was important for the success of this project.

## DISCLOSURE

The authors have no financial interest to declare in relation to the content of this article.

## Supplementary Material


