# Supplementary material for: Improving Accuracy and Timeliness of Nursing Documentation of Pediatric Early Warning Scores
Source: Pediatr Qual Saf. 2020 Mar 25;5(2):e278. doi: 10.1097/pq9.0000000000000278 (PMC7190257; doi:10.1097/pq9.0000000000000278)
Supplement: Supplementary file 1 [file pqs-5-e278-s001.docx]

Supplemental Digital Content

Improving Accuracy and Timeliness of Nursing Documentation of Pediatric Early Warning Scores

Nathan Dean

| **Pre Implementation** | **Post Implementation** |
| --- | --- |
| **PEWS Behavior**  🗌 0  🗌 1  🗌 2  🗌 3 | **Behavioral Subscore**  🗌 Playing/appropriate sleep pattern  🗌 Baseline behavioral activity  🗌 Increased sleepiness  🗌 Irritable/difficult to console  🗌 Increased baseline seizure activity  🗌 Lethargic  🗌 Confused |
| **PEWS Cardio**  🗌 0  🗌 1  🗌 2  🗌 3 | **Cardiac Subscore**  Heart Rate– insert value  Skin Color:  🗌 Pink  🗌 Pale  🗌 Mottled  Capillary Refill:  🗌 1-2 seconds  🗌 3 seconds  🗌 4 seconds  🗌 5-6 seconds  🗌 > 6 seconds |
| **PEWS Respiratory**  🗌 0  🗌 1  🗌 2  🗌 3 | **Respiratory Subscore**  Respiratory Rate– insert value  Respiratory Effort:  🗌 Normal  🗌 Mild increased WOB  🗌 Moderate increase WOB  🗌 Severe increased WOB  Oxygen Requirement:  🗌 Room air  🗌 Less than 40% FiO2  🗌 Less than 3 L/min NC  🗌 >40% FiO_2_ or > 3L/min NC  🗌 >50% FiO_2_ or > 4L/min NC |

SDC Table 1: Electronic health record pediatric early warning score documentation pre and post automated calculator implementation. *PEWS* pediatric early warning score, *WOB* work of breathing, *FiO_2_* Fraction of inspired oxygen, *L/min NC* liters per minute of nasal cannula

| Standardized Age-Based Vital Signs | | |
| --- | --- | --- |
| Age | Pulse (bpm) | Respirations (per minute) |
| 0 to < 6 months | 100-160 | 30-60 |
| 6 to <12 months | 110-160 | 24-38 |
| 1 to <3 years | 90-150 | 22-30 |
| 3 to <5 years | 80-125 | 22-30 |
| 5 to <10 years | 70-115 | 20-24 |
| 10 to <14 years | 60-100 | 16-22 |
| >/= 14 years | 60-100 | 14-20 |

SDC Table 2: Standardized Age Based Vital Signs for Pediatric Early Warning Score Calculation. *Bpm* beats per minute
